# Supplementary material for: Genomewide analysis of the Class III peroxidase gene family in apple (Malus domestica)
Source: PeerJ. 2025 Aug 18;13:e19741. doi: 10.7717/peerj.19741 (PMC12369634; doi:10.7717/peerj.19741)
Supplement: Supplemental Information 3 [file peerj-13-19741-s003.docx]

**The MIQE of qRT-PCR supporting file**

**1 The specificity of the primers**

The primers used in this study were blasted against Malus genome( taxi ID :3750), the blast results were shown below, the result showed that the primers were specific to the target genes.

PRX59 blast


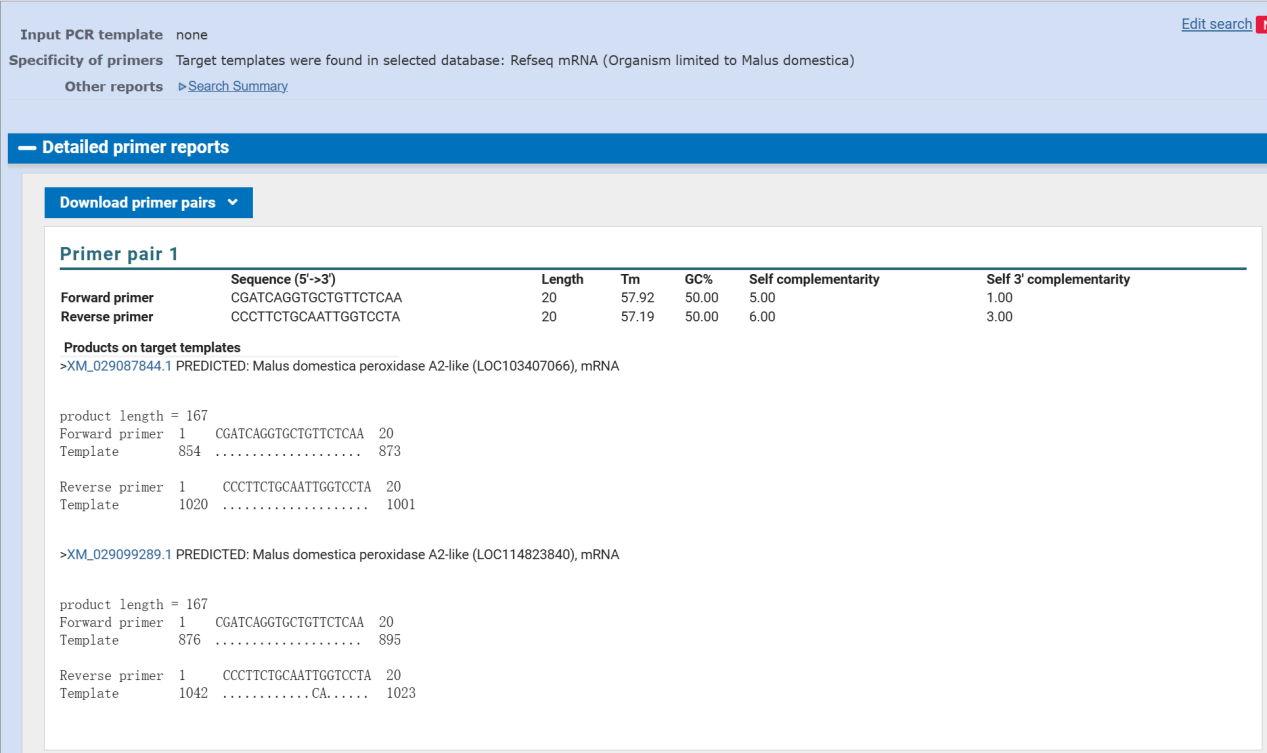


PRX87 blast


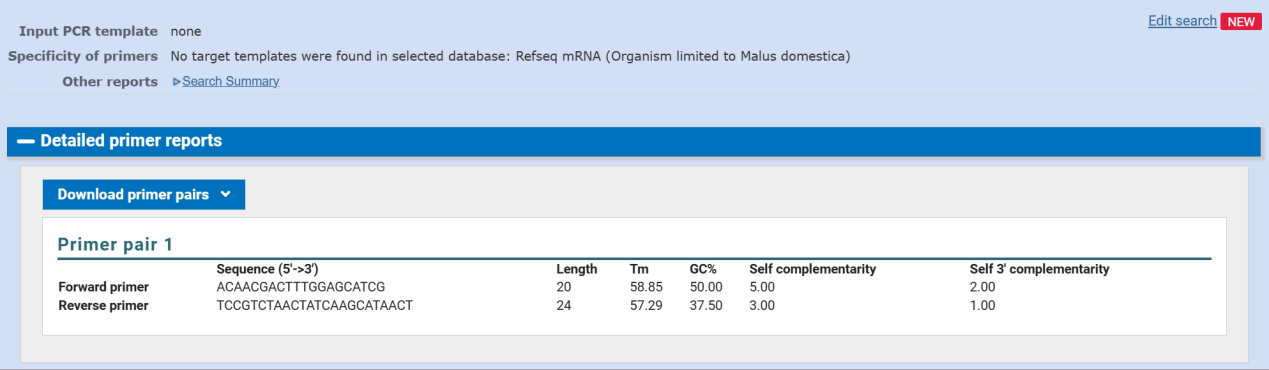


1. **The PCR amplication efficiency of primers used in this study**


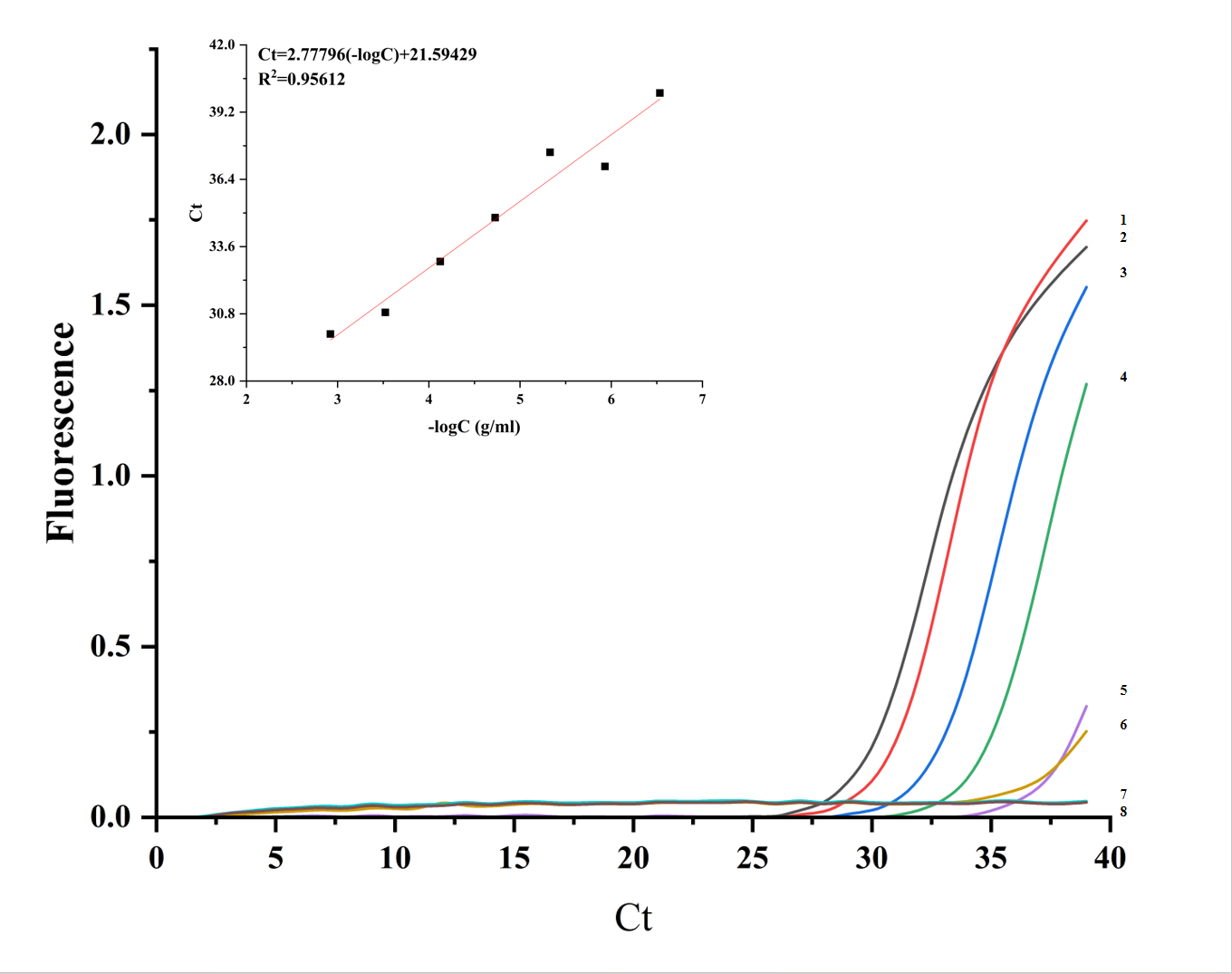


**MdPRX87**

Fig.1 The aplification curves , the slope and intercept of MdPRX87.1-7 curves represent a 4 folds series dilutions of DNA templates from 1200ng/μ l to 0.29ng/μl ,8 represent NTC, the results showed that the LOD of MdPRX86 gene is about 1.17ng/μl . A strong linear correlation was found between the logarithmic DNA concentration and the Ct values (R2 = 0.9561).


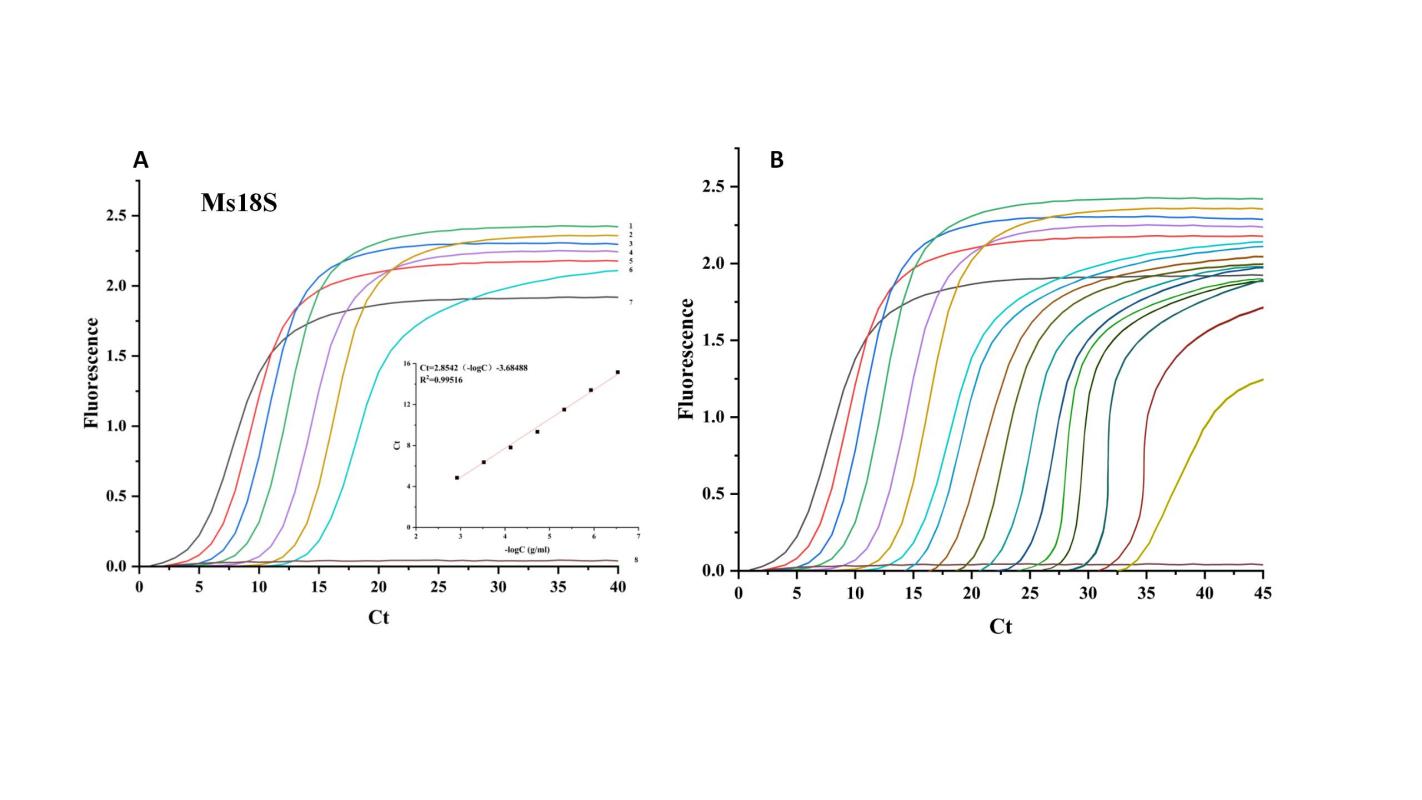


Fig.2 The amplification curves, correlation and detection limit of internal reference Md18S. A:The amplification curves and correlation equation of internal reference Md18S..1-7 curves represent a 4 folds series dilutions of DNA templates from 1200ng/μ l to 0.29ng/μl ,8 represent NTC, strong correlation was found between the logarithmic DNA concentration and the Ct values (R2 = 0.9951). B:The detection limit of Md18S, a series of 4-fold dilution of DNA templates from 1200ng/μl to 28 fg/μl, the results showed that LOD of Md18S is 28 fg/μl.
