# Supplementary figures and images for: Genomewide analysis of the Class III peroxidase gene family in apple (Malus domestica)
Source: PeerJ. 2025 Aug 18;13:e19741. doi: 10.7717/peerj.19741 (PMC12369634; doi:10.7717/peerj.19741)

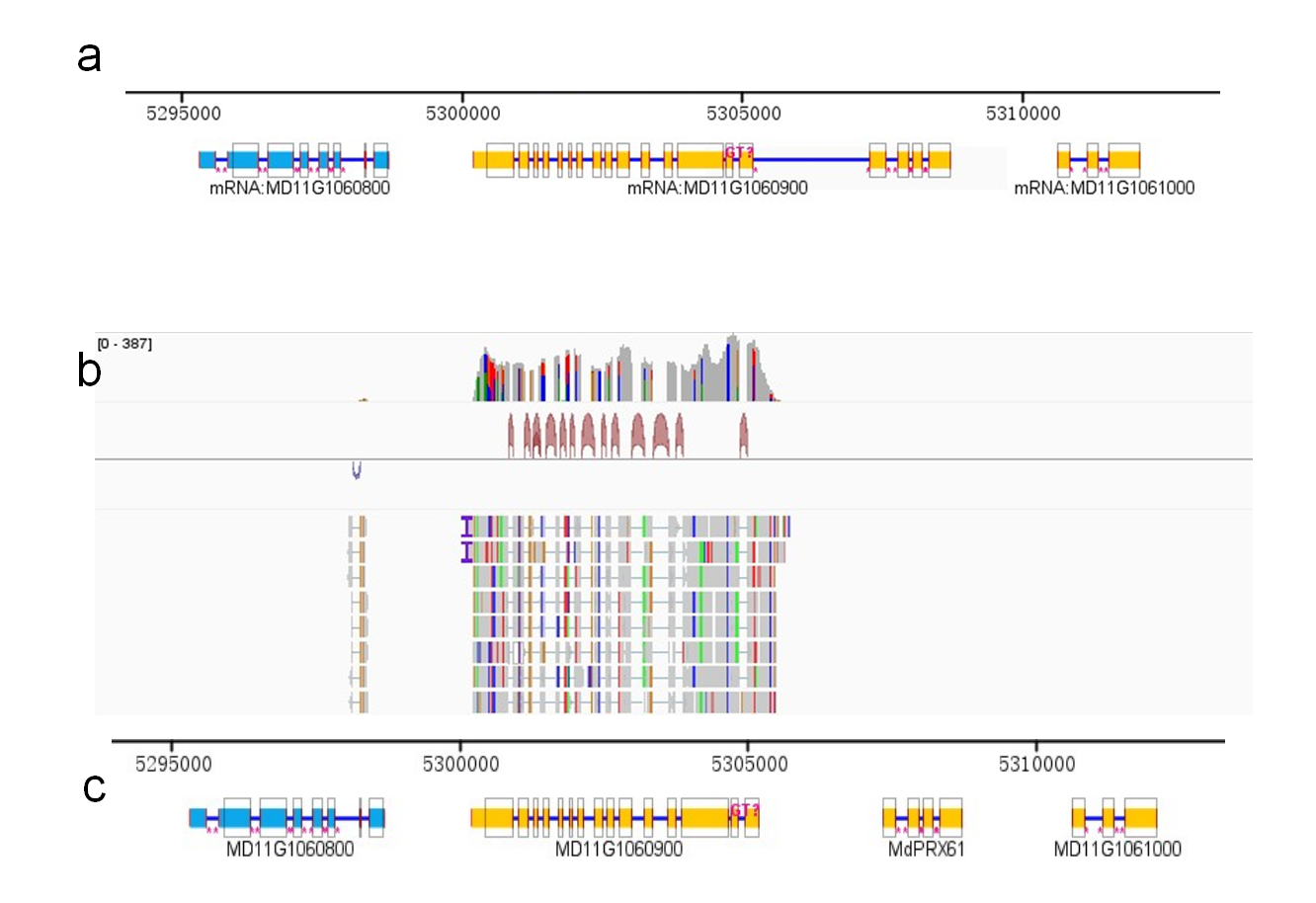

Supplement: Supplemental Information 9 — (a) the original annotation of MD11G106900 which predicted to be MdPRX61, it has 19 exons but there is no peroxidase domain in the first 15 exons (b) the RNA-Seq read mapping to mRNA MD11G106900 showed that the first 15 exons were expressed independently (c) the revised annotation of MdPRX61 by dividing the original MD11G106900 into two separate transcripts, the last four exons (16-19 exons) which contain a signal peptide and a peroxidase domains were renamed as MdPRX61. [file peerj-13-19741-s009.png]

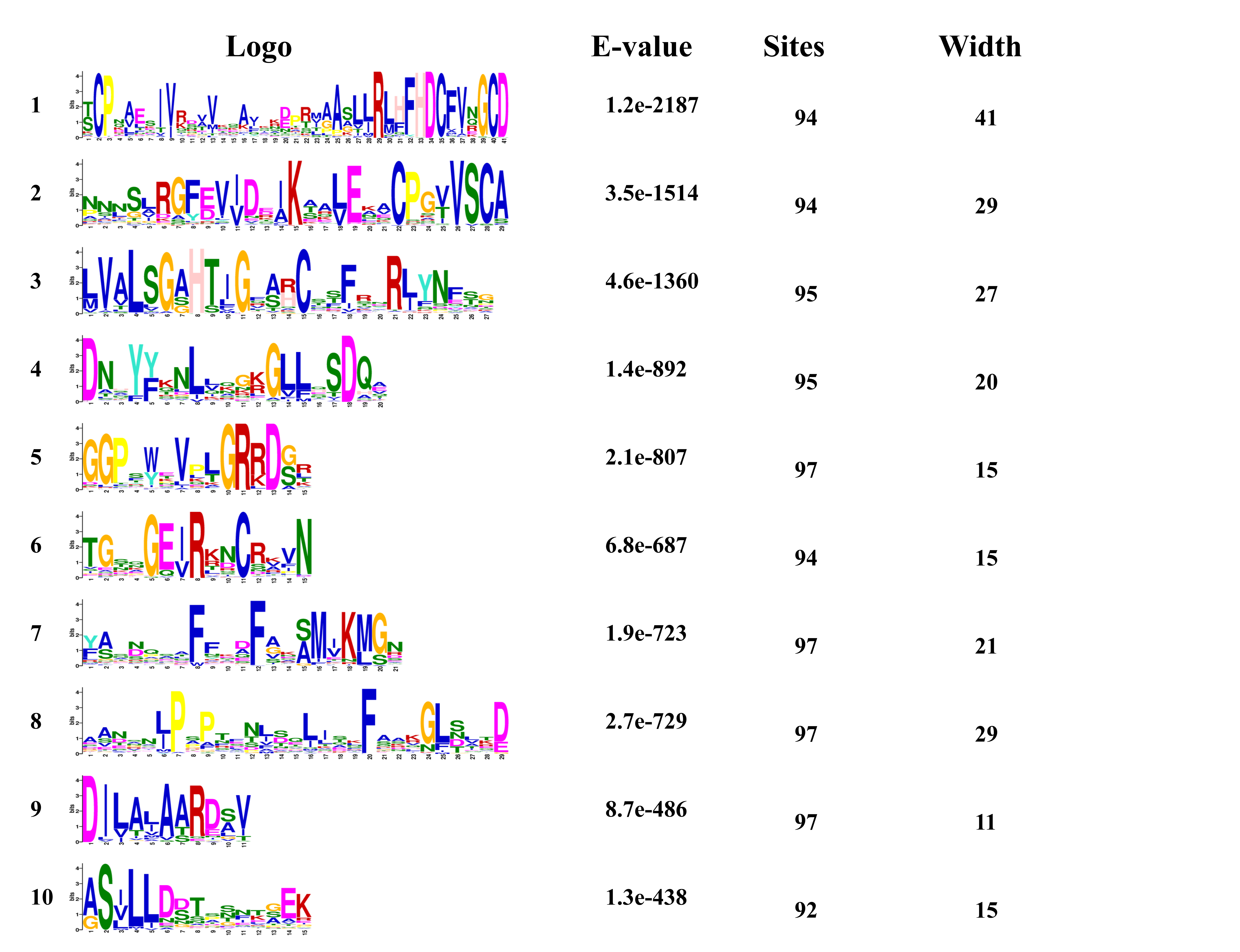

Supplement: Supplemental Information 10 [file peerj-13-19741-s010.png]

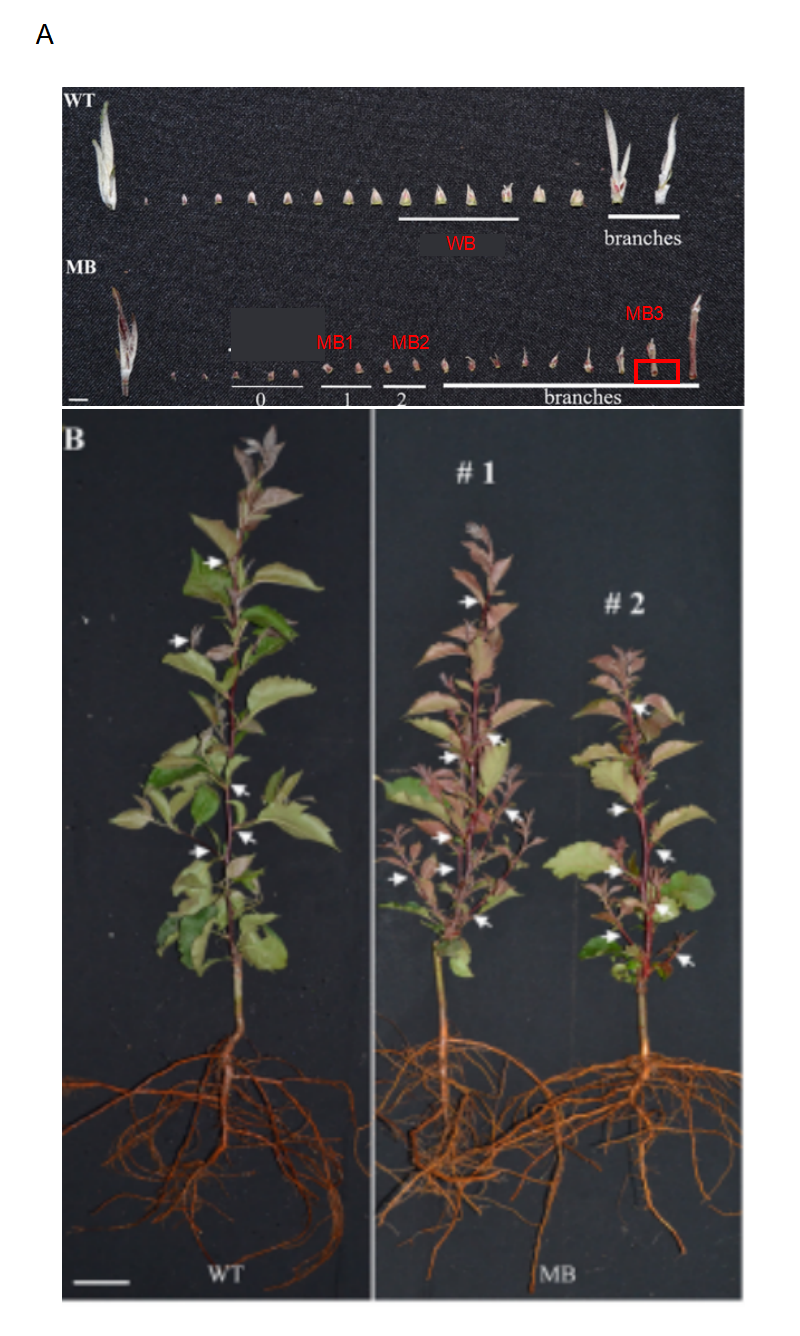

Supplement: Supplemental Information 11 — (A)Axillary bud phenotypes from the shoot apex to the branches in WT and MB at 60 DAB. The WB,MB1-MB3 indicates axillary buds and differentiated elongation zone of new branches used for RNA-seq and qRT-PCR. Scale bar = 5.0 mm. (B) Branching phenotypes of the WT and MB. Branching phenotype of the primary shoot of the WT and MB at 60 DAB. White arrows indicate the axillary buds or branches of partial nodes. #1 and #2 indicate different lines. [file peerj-13-19741-s011.png]
