# Supplementary material for: Genomewide analysis of the Class III peroxidase gene family in apple (Malus domestica)
Source: PeerJ. 2025 Aug 18;13:e19741. doi: 10.7717/peerj.19741 (PMC12369634; doi:10.7717/peerj.19741)
Supplement: Supplemental Information 12 [file peerj-13-19741-s012.doc]

**Table S1 Primer sequences**

| Primers | Sequences(5'to3'） |
| --- | --- |
| Mh18SF | ACACGGGGAGGTAGTGACAA |
| Mh18SR | CCTCCAATGGATCCTCGTTA |
| PRX59F | CGATCAGGTGCTGTTCTCAA |
| PRX59R | CCCTTCTGCAATTGGTCCTA |
| PRX86F | ACAACGACTTTGGAGCATCG |
| PRX86R | TCCGTCTAACTATCAAGCATAACT |
