# Supplementary material for: Genomewide analysis of the Class III peroxidase gene family in apple (Malus domestica)
Source: PeerJ. 2025 Aug 18;13:e19741. doi: 10.7717/peerj.19741 (PMC12369634; doi:10.7717/peerj.19741)
Supplement: Supplemental Information 13 [file peerj-13-19741-s013.doc]

**Table S2 The statistical data of MdPRX cis-elements**

| cis-element classification | numbers | percent |
| --- | --- | --- |
| Light responsive | 1113 | 0.6065 |
| MeJA-responsiveness | 237 | 0.1292 |
| Drought-responsive | 81 | 0.0441 |
| GA responsive | 74 | 0.0403 |
| ABA responsive | 69 | 0.0376 |
| Low-temperature responsive | 56 | 0.0305 |
| Auxin responsive | 43 | 0.0234 |
| Defense and stress | 40 | 0.0218 |
| Anaerobic induction | 39 | 0.0213 |
| Meristem expression | 38 | 0.0207 |
| Seed-specific regulation | 24 | 0.0131 |
| SA responsive | 21 | 0.0114 |
| Total | 1835 | 1 |
